# Synergistic Reactivation of Latent HIV Expression by Ingenol-3-Angelate, PEP005, Targeted NF-kB Signaling in Combination with JQ1 Induced p-TEFb Activation

**DOI:** 10.1371/journal.ppat.1005066

**Published:** 2015-07-30

**Authors:** Guochun Jiang, Erica A. Mendes, Philipp Kaiser, Daniel P. Wong, Yuyang Tang, Ivy Cai, Anne Fenton, Gregory P. Melcher, James E. K. Hildreth, George R. Thompson, Joseph K. Wong, Satya Dandekar

**Affiliations:** 1 Department of Medical Microbiology and Immunology, University of California, Davis, Davis, California, United States of America; 2 Department of Medicine, University of California, San Francisco, San Francisco, California, United States of America; 3 San Francisco Veterans Affairs Medical Center (VMAC), San Francisco, California, United States of America; 4 Department of Physics, Williams College, Williamstown, Massachusetts, United States of America; 5 Department of Molecular and Cellular Biology, University of California, Davis, Davis, California, United States of America; 6 Department of Internal Medicine, Division of Infectious Diseases, University of California Davis Medical Center, Sacramento, California, United States of America; Emory University, UNITED STATES

## Abstract

Although anti-retroviral therapy (ART) is highly effective in suppressing HIV replication, it fails to eradicate the virus from HIV-infected individuals. Stable latent HIV reservoirs are rapidly established early after HIV infection. Therefore, effective strategies for eradication of the HIV reservoirs are urgently needed. We report that ingenol-3-angelate (PEP005), the only active component in a previously FDA approved drug (PICATO) for the topical treatment of precancerous actinic keratosis, can effectively reactivate latent HIV *in vitro* and *ex vivo* with relatively low cellular toxicity. Biochemical analysis showed that PEP005 reactivated latent HIV through the induction of the pS643/S676-PKCδ/θ-IκBα/ε-NF-κB signaling pathway. Importantly, PEP005 alone was sufficient to induce expression of fully elongated and processed HIV RNAs in primary CD4+ T cells from HIV infected individuals receiving suppressive ART. Furthermore, PEP005 and the P-TEFb agonist, JQ1, exhibited synergism in reactivation of latent HIV with a combined effect that is 7.5-fold higher than the effect of PEP005 alone. Conversely, PEP005 suppressed HIV infection of primary CD4+ T cells through down-modulation of cell surface expression of HIV co-receptors. This anti-cancer compound is a potential candidate for advancing HIV eradication strategies.

## Author Summary

Stable latent viral reservoirs in HIV infected individuals are rapidly reactivated following the interruption of anti-retroviral therapy (ART). Despite an early initiation of ART, viral reservoirs are established and persist as demonstrated in the case of the Mississippi baby and from recent studies of the SIV model of AIDS. Therefore, new strategies are needed for the eradication of the latent HIV reservoirs. We found that ingenol-3-angelate (PEP005), a member of the new class of anti-cancer ingenol compounds, effectively reactivated HIV from latency in primary CD4+ T cells from HIV infected individuals receiving ART. Importantly, a combination of PEP005 and JQ1, a p-TEFb agonist, reactivated HIV from latency at level on average 7.5-fold higher compared to PEP005 alone. The potency of synergistic effects of PEP005 and JQ1 provide novel opportunities for advancing HIV eradication strategies in the future. In summary, ingenols represent a new group of lead compounds for combating HIV latency.

## Introduction

Anti-retroviral therapy (ART) is effective in suppressing HIV replication but it fails to eliminate latent viral reservoirs in HIV infected resting CD4+ T cells which, in blood, consist mainly of central and transitional memory CD4+ T cells [1–4]. Current ART options do not eradicate HIV from infected cells. In addition, these cells are invisible to the virus-specific immune responses in the setting of viral latency [5,6]. The viral reservoir is rapidly seeded and HIV latency might be established immediately after virus infection [7,8]. Despite initiation of ART in infants within hours of birth to HIV infected mothers, stable viral reservoirs were established and viral rebound occurred when therapy was interrupted [9]. In the simian immunodeficiency virus (SIV) model of AIDS, stable viral reservoirs are established within 2.5 days of infection [10]. The viral reactivation was detected in rhesus macaques following therapy interruption despite the initiation of ART at 3 days post SIV infection [10,11]. Collectively, these studies demonstrate that a very early initiation of ART may not be sufficient to prevent nor eliminate latent virus reservoirs [9,11,12]. It has been observed that the morbidity of HIV persistence in HIV-positive individuals on long-term ART includes drug toxicities and a higher risk of developing complications including dyslipidemia, cardiovascular disease and insulin resistance [13–15]. Therefore, a therapeutic cure of HIV is urgently needed that leads to viral eradication and experimental strategies for directly targeting HIV latent reservoirs are warranted.

Recent studies have explored an experimental strategy for viral eradication of HIV infected CD4+ T cells by activating HIV transcription and viral antigen expression from the latent viral reservoirs in the presence of ART [6]. This would lead to the detection and clearance of infected cells by the virus-specific host immune responses while the ART prevents new rounds of infection. Cytopathic effects of the viral reactivation would further increase the clearance of the latent viral reservoir. This “shock and kill” strategy was applied in a pilot clinical trial using the histone deacetylase (HDAC) inhibitor, vorinostat, in patients receiving suppressive ART [16–18]. The findings from these studies showed some promise but failed to result in significant clearance of residual HIV reservoirs. Potential mechanisms of this failure include the modest induction of HIV by this earlier generation of latency reversing agents (LRAs) used singly and due to immune defects in clearance of infected cells in spite of the reactivation of viral expression [19,20]. These studies demonstrate an urgent need for the development of new strategies both for disrupting HIV latency and facilitating elimination of infected cells after HIV expression is reactivated.

Several cell signaling pathways are critical for the establishment and maintenance of HIV latency [6,21,22]. Disruption of one or more of these pathways could lead to effective reactivation of HIV from latency. Various compounds have been tested for the disruption of HIV latency, and those inducing HIV reactivation from the viral long terminal repeat (LTR) through the stimulation of the protein kinase C (PKC)-NF-κB pathway showed high potency. These include phorbol esters (PMA and prostratin) and non-phorbol ester diterpenes (bryostatin and gnidimacrin) that induce NF-κB nuclear translocation and activation through the PKC pathway [22,23]. Some of these compounds effectively induce latent HIV reactivation *in vitro* at picomolar levels [24,25]. The LRAs, functioning through the PKC-NF-κB signaling, are able to reactivate latent HIV across a broad range of HIV latency models [20]. A recent study showed that LRAs stimulating PKC-NF-κB signaling may be most effective in inducing complete transcription of HIV from resting CD4+T cells of HIV infected individuals on suppressive ART [26]. Moreover, these compounds cause down-modulation of the expression of cell surface receptors, CD4, CXCR4 or CCR5, and protect cells against HIV infection [22]. Therefore, LRAs that activate PKC-NF-κB signaling are potential candidates for HIV cure studies. We previously reported that an ingenol ester, ingenol-3-hexanoate or IngB, is an excellent candidate for the reactivation of HIV from latency [24]. The modified ingenol-3-hexanoate was originally isolated from an Amazonian plant, *Euphorbia tirucalli*. It exerts low toxicity in CD4+ T cells and does not induce global T-cell activation. It caused reactivation of latent HIV at nanomolar levels [24]. However, since IngB induces expression and activation of both NF-κB and CyclinT1/CDK9, and stimulate IFNγ expression in primary CD4+ T cells, further search for new ingenol compounds with better HIV reactivation potential and lower cellular toxicity is needed [24,27,28].

Among previously identified ingenol compounds, ingenol-3-angelate (PEP005) is currently approved for clinical use. A recently FDA approved drug, PICATO, for the topical treatment of precancerous actinic keratosis contains ingenol-3-angelate as an active component [29]. A prior study suggested that ingenol-3-angelate could induce HIV expression from the U1 monocyte cell line harboring HIV genome [30]. In the current study, we report that PEP005 can effectively reactivate latent HIV through the activation of the pS643/S676-PKCδ/θ-IκBα/ε-NF-κB pathway in an HIV latency model *in vitro* but does not induce or increase NF-κB protein production by itself. It also reactivated full-length HIV transcription based on an assay targeting the poly A tail region of HIV transcripts in cells from ART-suppressed HIV-positive individuals, while exerting minimal toxicity and effects on T cell activation *ex vivo* [26]. Importantly, the effect of PEP005 was synergistic with JQ1, a p-TEFb activator, and the combination was highly potent in reactivating latent HIV expression both *in vitro* and *ex vivo*. Our findings identify this anti-cancer drug, PEP005, as having a distinct mechanism of molecular signaling and as a potential candidate for advancing to future HIV eradication studies.

## Materials and Methods

### Cell culture

J-Lat A1 cells (harboring a single copy of latent HIV LTR and one copy of green fluorescent protein gene under the HIV LTR control) or U1 cells (harboring two latent HIV genomes with defective Tat gene) were cultured in RPMI1640 medium with 10% fetal bovine serum (FBS) and 1% Pen/strep in a 37°C incubator containing 5% CO_2_ [24,31]. Both of the cell lines were obtained from NIH AIDS Reagent Program. For reactivation of HIV LTR, cells were treated with PMA (Sigma), JQ1 (Biovision), Prostatin (Sigma), SAHA (Santa Cruz), TNF-α (BD), GSK343 (Sigma), or PEP005 (Tocris Bioscience) for 24h. HIV reactivation was quantified by GFP expression using flow cytometry and the data were analyzed using FlowJo Software for J-Lat A1 cells, or by quantitative RT-PCR (RT-qPCR) for J-Lat A1 and U1 cells. Cell viability was evaluated using Live/Dead dye (Life Technologies) by flow cytometry. So far, there is not a single *in vitro* cell culture model available that captures all the features of HIV latency. However, PKC agonists seem to reactivate latent HIV in all the cell culture models of viral latency. Similar to other J-Lat cell lines, the J-Lat A1 clone has been widely used by researchers for HIV latency studies. The T-cell-derived J-Lat A1 cells harbor one copy of a construct containing a Tat and a green fluorescent protein (GFP) gene framed by the 5’ and 3’ HIV LTR [32]. The J-Lat A1 cells also contain the TAR loop located within the R region of HIV LTR (nt +1 to +60), which provides an opportunity for Tat-TAR interactions for the modulation of transcription and transcriptional elongation. Consequently, the J-Lat A1 cell model is a suitable cell culture model to investigate latency reversal by PKC agonists.

### Primary CD4+ T cell isolation and detection of T cell activation markers

Peripheral blood samples were collected from 13 HIV-infected individuals receiving suppressive ART for >3 years except for one patient. All subjects had suppression of plasma viremia for more than 6 months (Average 5.84 years). At the time of the study enrollment, CD4+ T cell counts in peripheral blood samples ranged from 264 to 1100 cells/mm^3^ (Average 639 cell/mm^3^) and plasma viral loads were <20 copies per ml as measured by qPCR (**Table 1**). Isolation of the peripheral blood mononuclear cells (PBMC) and purification of CD4+ T cells using the EasySep kit (STEMCELL Technologies Inc. Vancouver, BC, Canada) were performed as previously described [24]. The purified CD4+ T cells were plated at a density of 1x10^6^ cells/ml and treated with 200 ng/ml PMA plus 2 μM Ionomycin, 6–12 nM PEP005, 2 μM of JQ1, or 12 nM PEP005 plus 2 μM JQ1 for 6 hrs or 48 hrs and the cells were collected for RNA purification. To measure changes in the cell activation status of CD4+ and CD8+ T cell subsets, PBMCs were isolated from uninfected controls and 2x10^6^ cells were incubated with DMSO, 200 ng/ml PMA plus 2 μM Ionomycin, 6–12 nM PEP005 for 24 hrs or 72 hrs, and immunostained with anti-CD3, anti-CD38, anti-CD69, or anti-HLA-DR antibodies (Biolegend) for 20 min at 4°C. Cells were fixed in 1% PFA and analyzed by flow cytometry (FlowJo software from TreeStar). In addition, PBMCs from HIV-negative uninfected controls were similarly treated for 24 or 72 hrs and cells were collected for cytokine analysis using RT-qPCR.

**Table 1:** Characteristics of HIV-positive individuals receiving HAART.

Patients	Age	Sex	Viral load	CD4 count	ART	Time under ART(years)
**2040**	45	M	<20	347	Tenofovir/Emtricitabine /Raltegravir	4
**2041**	56	M	<20	1095	Emtricitabine/tenofovir /efavirenz	7
**2044**	60	F	<20	1100	Emtricitabine/tenofovir /efavirenz	8
**2050**	48	M	<20	757	Emtricitabine/tenofovir /efavirenz	8
**2051**	56	M	<20	368	Emtricitabine/tenofovir /efavirenz	5
**2052**	35	M	<20	264	Lopinavir/Ritonavir	4
**2053**	50	M	<20	260	Atazanavir/Ritonavir	6
**2054**	53	M	<20	477	Emtricitabine/tenofovir /efavirenz	12
**2055**	45	M	<20	736	Tenofovir/Emtricitabine /Atazanavir/Ritonavir	6
**2056**	63	M	<20	510	Nevirapine/Tenofovir /Emtricitabine	5
**2057**	55	M	<20	775	Tenofovir/Emtricitabine /Atazanavir/Ritonavir	3
**2060**	57	M	<20	813	Atazanavir/Ritonavir /Tenofovir/Emtricitabine	6
**2061**	57	M	<20	805	Elvitegravir/Cobicistat/ /Emtricitabine/Tenofovir	2

### Cell viability and proliferation measurements

Cells were placed in 96-well plates and incubated for 24 or 72h hrs with compounds. Cell viability was measured using MTT assay (Roche Laboratories), and cell proliferation/S-phase progression was determined by using BrdU ELISA kit (Cell signaling, #1863).

### Immunoblot analysis

One million J-Lat A1 cells or PBMCs from HIV-negative uninfected controls were incubated with 12 nM PEP005 for 6 hrs. Whole cell protein extracts were prepared with RIPA buffer containing proteinase inhibitors and phosphatase inhibitors (Sigma). Expression of the isoforms of PKC protein or NF-κB/p65 was evaluated using the PKC Isoform Sampler Antibody Kit (Cell Signaling, 9960S) or anti-NF-κB/p65 (Abcam). The level of phosphorylation of PKC was determined using anti-Phospho-Ser664-PKCδ, anti-Phospho-Ser643/676 PKCδ or anti-Phospho-T538-PKCθ (Millipore), and the level of phosphorylation of IκB was determined using p-IκBα (Ser32), p-IκBβ (Thr19/Ser23) or p-IκBε (Ser18/22) antibodies (Millipore).

### HIV RNA quantification in patient samples

Total RNA was extracted using the Qiagen RNeasy Kit that included a DNA digestion step. Quantitative RT-PCR was performed using Taqman Fast Virus 1-Step Master Mix (Applied Biosystems) in a ViiA7 real-time PCR system (Applied Biosystems). Transcripts containing the U5 region of the 5’ LTR were amplified using well-conserved primers (HXB2 559–543, 626–643) and fluorescent probe (HXB2 559–584) [24,33]. Transcripts containing the U3 to R region of the 3’ LTR including a Poly A tail were amplified in a one-step RT-PCR procedure related to a recently published assay with primers and probe binding sites as follows: gccctcagatgctrcatataa (HXB2 9496–9516), ttttttttttttttttttttttttttgaag (9632–9636 + poly T) and FAM-tgcctgtactgggtctctctggttag-MGB (HXB2 9529–9554) [26,34]. External HIV RNA standards were prepared from *in vitro* transcripts quantified by spectrophotometry. HIV RNA copy numbers were normalized to RNA input.

### Quantitative analysis of synergy of latency reversing agent combinations

We adapted the Bliss independence model as implemented by Laird et al to test for synergy when PEP005 was combined with other latency reversing agents [35,36]. For drugs x and y, we used the equation *fa*_*xyP*_ *= fa*_*x*_ *+fa*_*y*_ *−(fa*_*x*_ *)(fa*_*y*_ *)*, where *fa*_*xyP*_ represents the predicted fraction affected by the combination of drug x and drug y given the observed effects of drug x (*fa*_*x*_) and drug y (*fa*_*y*_) used individually and *fa*_*xy*,*O*_ = the observed effect when x and y were tested together. Calculation of *fa*_*x*_ for U1 cells and patient derived T cells followed the approach of Laird for intracellular HIV RNA: *fa*_*x*_ *= (HIV RNA copies with drug x–background copies with DMSO)/ (HIV RNA copies with PMA–background copies with DMSO)*. For these analyses, we included data for which every parameter for the synergy analysis was available and excluded individual cases where some of the parameters were not available. In cases where one or more experimental drug conditions resulted in RNA expression exceeding the PMA condition, we imputed the highest HIV RNA value in that experiment +1 to represent the denominator for calculation of *fa*_*x*_. The calculation of *fa*_*x*_ for J-Lat A1 cells used the % GFP positive cells in place of intracellular HIV RNA. With this model, Δ *fa*_*xy*_ *= fa*_*xyO*_ (the observed fraction affected by the drug combination)—*fa*_*xyP*_ (the predicted fraction affected by the drug combination) provides an indication of synergy (Δ *fa*_*xy*_ > 0), additive effect (Bliss independence) (Δ *fa*_*xy*_ = 0) or antagonism (Δ *fa*_*xy*_< 0). Calculations analyzing synergy were done using Python. Statistical significance was determined using a one tailed ratio t-test executed in the statistical package “R”.

### Chromatin immunoprecipitation (ChIP)

ChIP assay was performed as previously described [24,37]. Briefly, 1X10^6^ J-Lat A1 cells were incubated with PEP005, or PKCδ/θ inhibitor (PKCδi, Millipore/Calbiochem), fixed in 1% formaldehyde then resuspended in lysis buffer containing 1% SDS, 10 mM EDTA, 50 mM Tris-HCl, pH 8.1 (ChIP Assay Kit, Millipore) and protease inhibitor cocktail (Sigma-Aldrich). Lysates were sonicated to obtain DNA fragments of 200–1500 bp. The immune complex was retrieved by incubating for 45 min with 50 μl of protein A/G-agarose beads saturated with BSA/salmon sperm DNA. Following the washes, the chromatin was eluted and reverse cross-linked overnight. DNA was extracted (Qiagen PCR purification kit) and quantitative real-time PCR was performed using Agilent Brilliant Ultra-Fast SYBR Green QPCR reagent using the 7500 real-time PCR System. The upstream primer sequence was 5’-AGCTTGCTACAAGGGACTTTCC-3’, and the downstream primer sequence was 5’-ACCCAGTACAGGCAAAAAGCAG-3’.

### Real-time PCR analysis of GFP or HIV gene expression

Total RNA was isolated from J-Lat A1 cells or U1 cells using the RNeasy Kit (Qiagen) followed by digestion with DNase I (Invitrogen). First strand cDNA was synthesized using Superscript II (Invitrogen). Real-time PCR (TaqMan) was performed on a ViiA 7 detector using the following primer/probe set: for J-Lat A1 cells, primer 1: 5’-GGAGCGACCATCTTCTTCA-3’, primer 2: 5’-AGGGTGTCGCCCTCGAA-3’, probe 5’-FAM CTACAAGACCC GCGCCGAGGTG TAMRA-3’, for U1 cells HIV 5’ LTR primers/probe were used (see above) [24]. The GAPDH primer/probe set was purchased from Applied Biosystems.

### Pre-treatment of primary CD4+ T cells with PEP005 and HIV-1 infection of primary CD4+ T cells

Primary CD4+ T cells were isolated from peripheral blood samples of healthy HIV-negative donors and were pre-treated with PEP005 overnight. The CD4+ T cells were infected with HIV-1 (HIV-1 IIIB expanded in Jurkat T cells, 100ng p24-gag) through spinoculation as described previously [38]. Following an exposure of 24 hrs to HIV, the cells were washed. Cell supernatants were collected and HIV p24 levels were measured by ELISA. The cells were also collected for RNA extraction and measurement of viral transcripts by RT-qPCR.

### Ethics statement

Studies were performed under informed written consent and a protocol approved by the University of California-Davis Institutional Review Board (IRB 219139–7).

### Statistical analysis

Means and standard errors (SE) were calculated for all data points from at least 3 independent experiments in triplicates. Statistical significance was determined using the two-way Student t test, where p value<0.05 considered significant.

## Results

### PEP005 induces HIV expression in an HIV latency cell culture model *in vitro*

**Fig 1:**
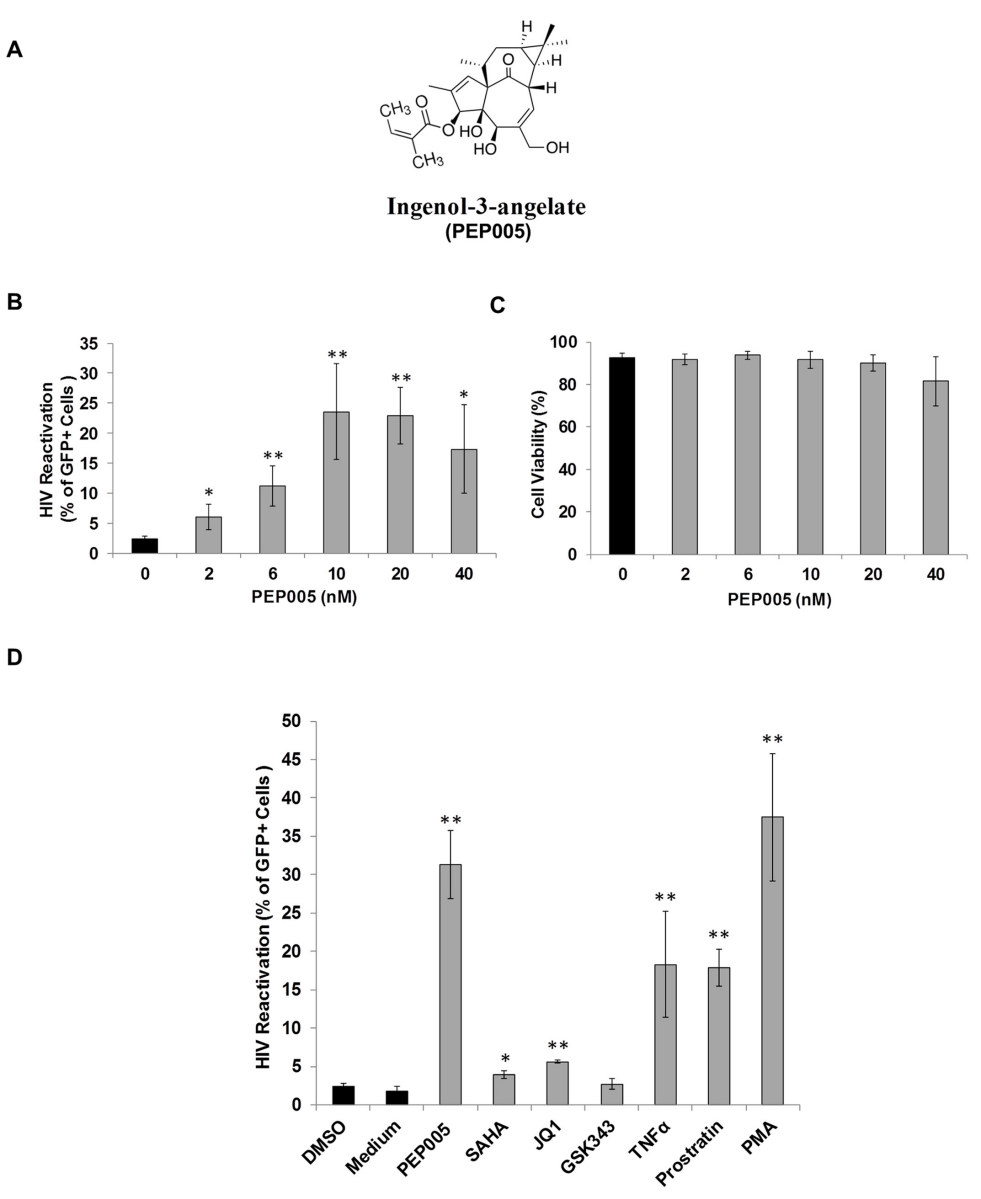
PEP005 induces reactivation of HIV latency *in vitro*. J-Lat A1 cells were exposed to different concentrations of PEP005 and virus reactivation was measured by GFP expression using flow cytometry. Cell viability was determined by Live/Dead dye staining. **(A)** Chemical Structure of PEP005. **(B, C)** PEP005 reactivates HIV in a dose-dependent manner and displays minimal cytotoxicity. **(D)** PEP005 potently reactivates latent HIV in cell lines compared with other LRAs. J-Lat A1 cells were treated with 10 nM PEP005, 500 nM SAHA, 2 μM JQ1, 2 μM GSK343, 10 ng/ml TNF, 2 μM Prostratin, or 5 ng/ml PMA for 24 hours and the percentage of GFP expression was evaluated using flow cytometry. *, p<0.05; **, p<0.01.

In order to determine the potential of PEP005 to induce HIV expression, J-Lat A1 cells, an established HIV latency lymphocyte cell culture model *in vitro* [24,32,39], were treated with increasing concentrations (2–40 nM) of PEP005 **(Fig 1A)**. PEP005 induced HIV expression in a dose-dependent manner and in the absence of any apparent cellular toxicity. A 7-fold increase in the reactivation of HIV latency was detected in J-Lat A1 cells at 20 nM of PEP005 as compared to untreated controls (**Fig 1B and 1C**). The effect of PEP005 on HIV expression was evident even at the 2 nM level. Compared to other compounds known to reactivate HIV from latency, PEP005 appeared to be more potent than SAHA (a histone deacetylase inhibitor), JQ1 (a BET bromodomain inhibitor) and GSK343 (an inhibitor of EZH2) (**Fig 1D**). At the 10 nM level, PEP005-induced HIV reactivation was similar to that induced by PMA and more potent than 2 μM Prostratin (p = 0.012). Interestingly, although EZH2 was shown to be critical for establishment of HIV latency through tri-methylation of H3K27 and inhibition of EZH2 by 3-deazaneplanocin A (DZNep) resulted in reactivation of latent HIV *in vitro* [40], the specific EZH2 inhibitor, GSK343 (recently developed by GSK) [41,42], failed to induce HIV expression in J-Lat A1 cells (**Fig 1D**). Taken together, these data show that PEP005 is highly potent in reactivating latent HIV *in vitro*.

### The effect of PEP005 on reactivation of latent HIV is potently enhanced by combination with JQ1 in both J-Lat A1 cells and U1 cells *in vitro*

**Fig 2:**
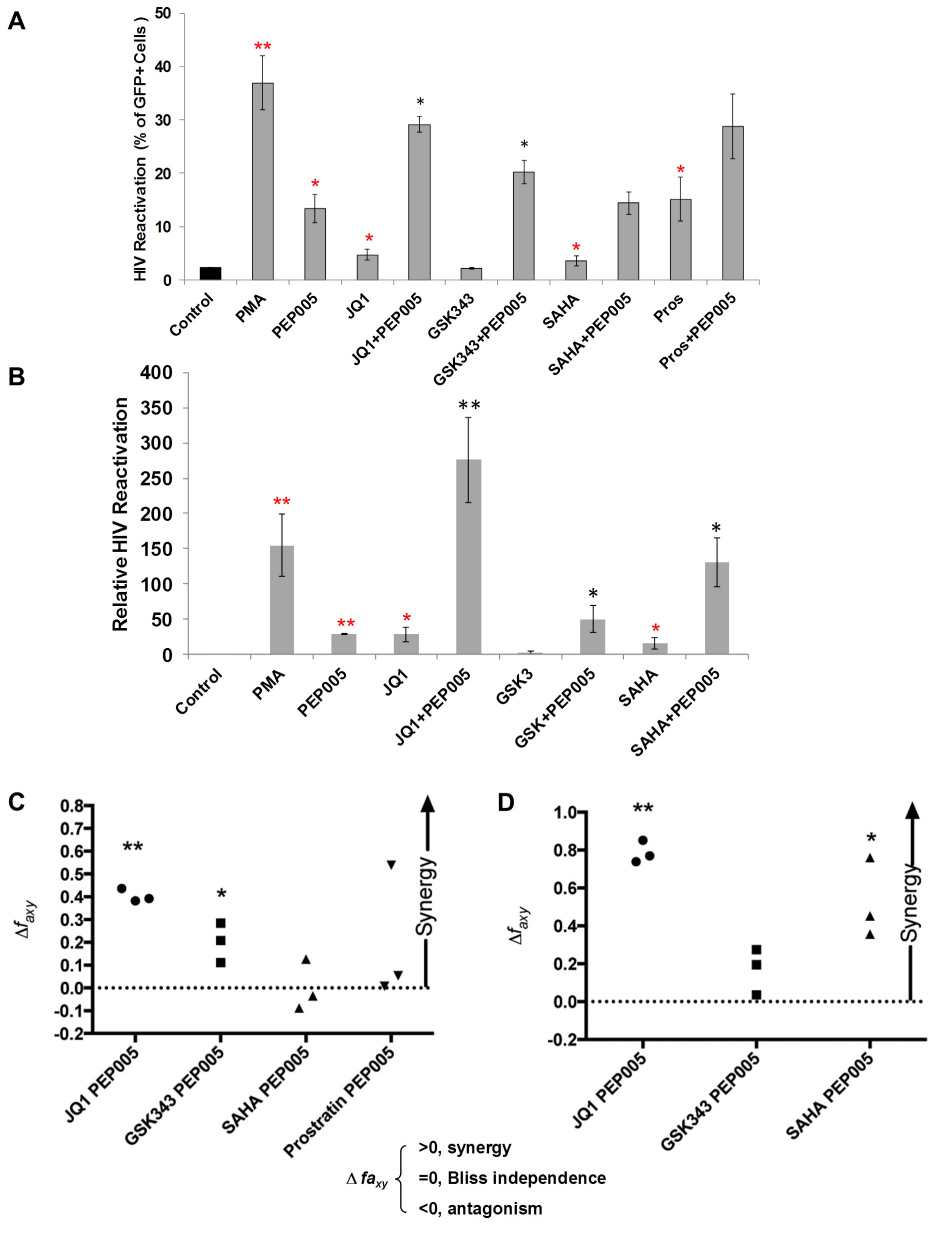
PEP005 synergizes with other latency reversing agents in reactivating latent HIV. **(A)** J-Lat A1 cells were treated with 5 ng/ml PMA, 6 nM PEP005, 500 nM SAHA, 2 μM JQ1, 2 μM GSK343, or 10 μM Prostratin alone or in combination with 6 nM PEP005 for 24 hours and the percentage of GFP expressing cells was determined using flow cytometry. **, p<0.01; *, p<0.05 compared with control treatment; **, p<0.01; *, p<0.05 compared with PEP005 treatment alone. **(B)** The U1 cells were treated with 5 ng/ml PMA, 6 nM PEP005, 500 nM SAHA, 2 μM JQ1, or 2 μM GSK343, alone or in combination with 6 nM PEP005 for 24 hours and the HIV transcription was evaluated using RT-qPCR. Numbers indicate fold-increase over the control. **, p<0.01; *, p<0.05 compared with control treatment; **, p<0.01; *, p<0.05 compared with PEP005 treatment alone. **(C** and **D)** PEP005 synergizes with other LRAs to significantly increase GFP or HIV-1 mRNA expression in J-Lat A1 **(C)** or U1 **(D)** cell lines. The Bliss independence model was utilized for calculation of synergy for LRA combinations [35] (See materials and method). Dotted horizontal line signifies pure additive effect (∆f_axy_ = 0). Synergy is defined as ∆f_axy_>0 while ∆f_axy_<0 indicates antagonism. Statistical significance was determined using a one tailed ratio t-test comparing predicted and observed drug combination effects. *p < 0.05; **p < 0.01.

Several molecular pathways are involved in the establishment and maintenance of HIV latency [6,21]. In order to optimally reactivate latent HIV expression, we utilized combinations of latency reversing agents (LRAs) in the J-Lat A1 cells and U1 cells *in vitro* [43]. Several previously tested compounds were selected including the HDAC inhibitor, SAHA (500 nM); the BET bromodomain inhibitor, JQ1 (2 μM); the EZH2 inhibitor, GSK343 (2 μM); and the PKC agonist Prostratin (10 μM), in combination with PEP005 (6 nM). A lower concerntration of PEP005 was used in these assays since PEP005 is very potent in reactivating latent HIV expression (**Fig 1D**) and the combined effects of it with other LRAs would be difficult to distinguish at a higher concentration of PEP005 [24]. PEP005 induced reactivation of latent HIV and was highly effective in combination with JQ1 in J-Lat A1 cells (**Fig 2A**). Surprisingly, GSK343 alone could not effectively induce HIV expression but was able to enhance the magnitude of latent HIV reactivation by PEP005. Our findings suggest that pre-disruption of H3K27Me3-mediated chromatin repression may be required for enhanced latent HIV reactivation. Interestingly, Prostratin also showed a trend for enhancing effect of PEP005 on latent HIV reactivation. However, this was not statistically significant.

To determine whether the combined effects of PEP005 on latent HIV reactivation occurs in other latent HIV-infected immune cell types, we examined HIV transcription in U1 cells, a well-studied promonocyte cell line that harbors two complete HIV genomes with *Tat* gene mutations and is used as an HIV latency cell culture model [31]. While PEP005 alone induced about 25-fold increase in latent HIV reactivation compared to controls, addition of JQ1 boosted induction of HIV transcription to more than 250-fold increase (**Fig 2B**). We found a combined effect of PEP005 on reactivation of HIV latency with GSK343, as well as with SAHA in U1 cells. We previously reported a similar pattern of synergy when J-Lat A1 cells were treated with IngB and JQ1 [24].

Our data indicated that PEP005 might synergistically reactivate latent HIV in both J-Lat A1 cells and U1 cells. To assess whether these combined effects meet criteria for drug synergy, we compared the experimentally observed combined effects in J-Lat A1 cells and U1 cells to the effects predicted under the Bliss independence model for combined drug effects ([35] and **Fig 2C and 2D**). This model assumes that if two compounds act through different mechanisms, their effects are merely additive in the absence of synergistic interactions. In contrast, effects of combinations that are greater or lesser than the idealized Bliss independence prediction imply synergy or antagonism respectively [31]. We found that PEP005 demonstrates significant synergism with JQ1 or EZH2 inhibitors to induce GFP expression *in vitro* (**Fig 2C**). HDAC inhibitor SAHA and another PKC agonist Prostratin did not exhibit synergy with PEP005. In U1 cells, PEP005 synergized significantly with JQ1 or SAHA to induce HIV mRNA expression *in vitro* (**Fig 2D**). But GSK343 did not exhibit synergy with PEP005. Taken together, PEP005 exerted a synergic effect with JQ1 on reactivation of HIV from latency in both in J-Lat A1 and U1 cell lines.

### PEP005 disrupts HIV latency through IκBα/ε-pSer664/Ser676-PKCδ/θ-NF-ĸB signaling

**Fig 3:**
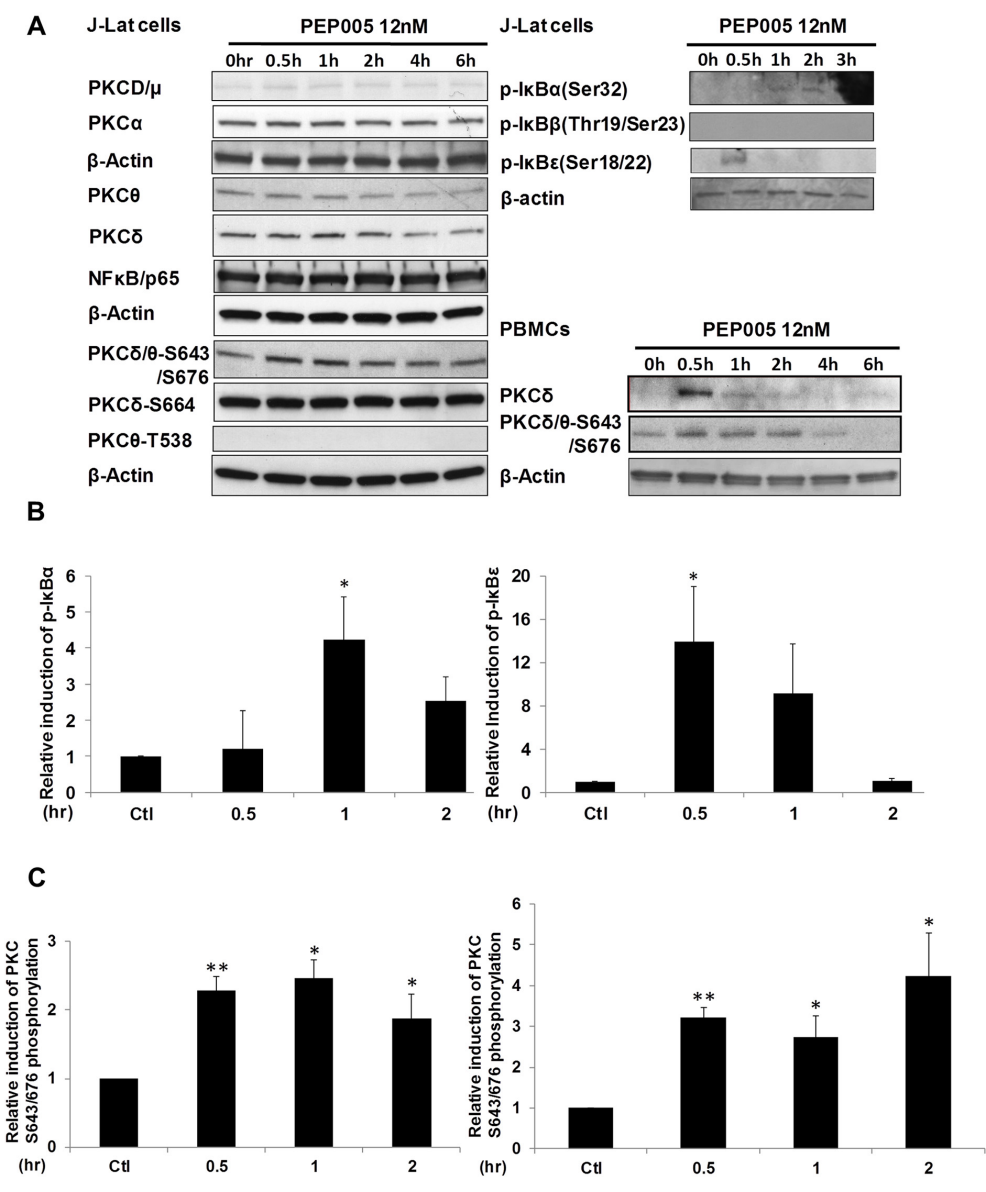
PEP005 activates PKCδ/θ-IκBα/ε-NF-ĸB signaling. **(A)** J-Lat A1 cells or PBMCs isolated from peripheral blood of healthy HIV-negative individuals were treated with 12 nM of PEP005 for up to 6 hours. Western blot analysis was performed to detect the expression of PKC isoforms, IκB isoforms, as well as expression of NF-κB/p65. **(B)** Quantitation of phosphorylation of IκBα or IκBε in J-Lat A1 cells after 2hr treatment with PEP005 in panel A. Relative band intensities from three independent experiments in J-Lat A1 cells as determined using ImageJ (NIH) are shown in the bar graph. * p<0.05. **(C)** Quantitation of PKCδ/θ S643/S676 phosphorylation after 2hr treatment of PEP005 in panel A. Relative band intensities from three independent experiments in J-Lat A1 cells (Left panel) or PBMCs (Right panel) as determined using ImageJ (NIH) are shown in the bar graph. * p<0.05, ** p<0.01.

**Fig 4:**
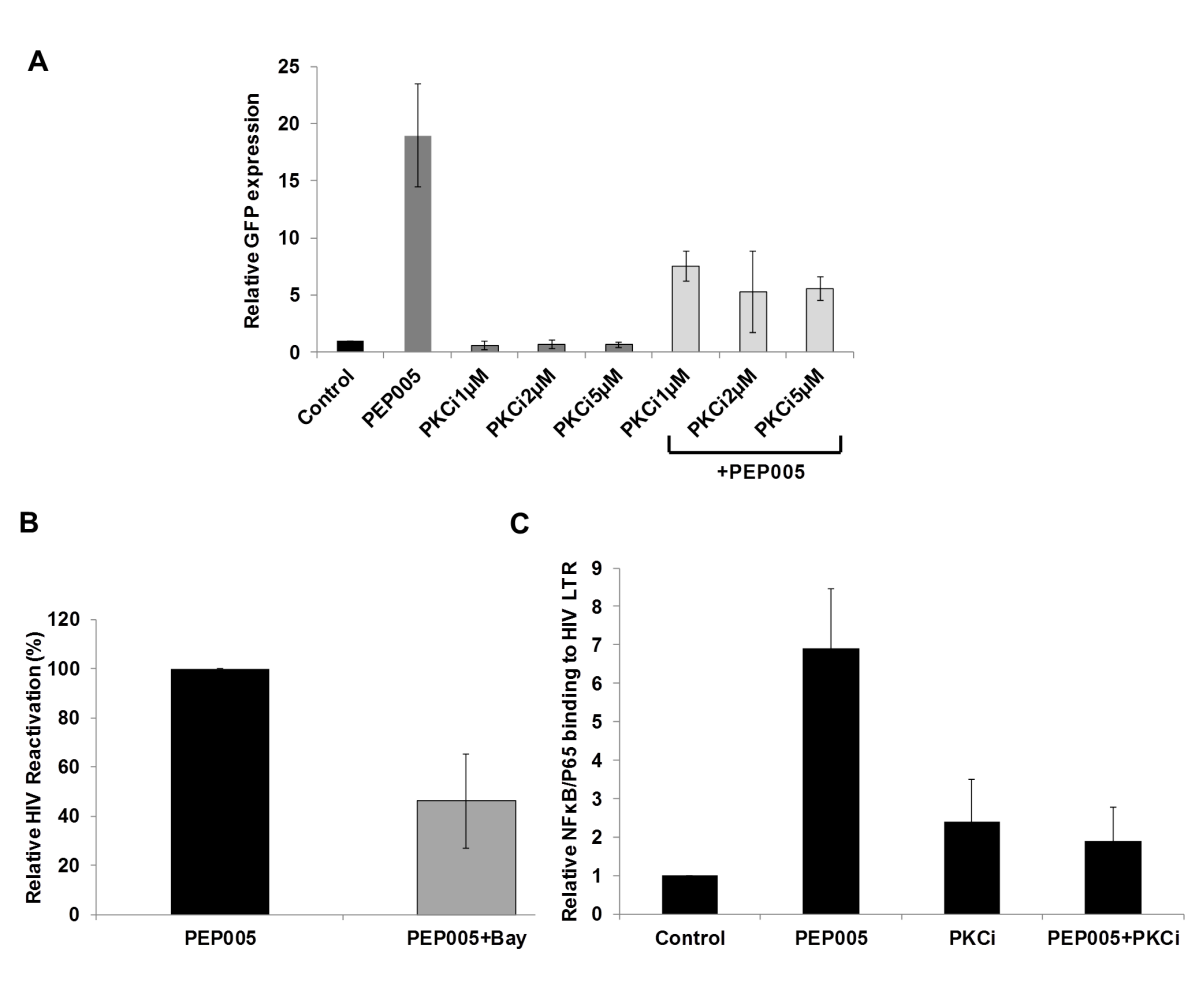
PEP005-induced HIV reactivation is mediated through PKCδ/θ-IκBα/ε-NF-ĸB signaling. **(A)** PEP005-induced HIV reactivation is suppressed by inhibition of the PKCδ/θ. J-Lat A1 cells were treated with 6 nM of PEP005 in the presence of 1, 2 or 5 μM of PKC inhibitor (PKCθ/δ inhibitor; Milipore/Calbiochem (539649)) and evaluated for GFP expression by RT-qPCR. **(B)** NF-ĸB inhibition partially suppresses PEP005-induced HIV reactivation in J-Lat A1 cells. J-Lat A1 cells were treated with 6 nM of PEP005 in the presence of 2.5 μM Bay 11–7082, an NF-ĸB inhibitor, and were evaluated for GFP expression by RT-qPCR. **(C)** J-Lat A1 cells were treated with PEP005 or alternatively with PKC inhibitor alone or in the presence of PEP005 and the relative binding of NF-ĸB to the HIV LTR was determined using ChIP-qPCR.

Although it was shown that PEP005 can activate the PKC-NF-κB pathway, it is not known at which step of the PKC-NF-κB pathway is modulated during the reactivation of latent HIV. Therefore, we examined protein expression of several components of the PKC pathway in J-Lat A1 cells by Western blot analysis using antibodies specific for four PKC super families, including PKCμ/D, PKCα, PKCδ, and PKCθ. There was no significant induction of expression of these PKC proteins except for a modest up-regulation of PKCδ that was detected at 1 hr following 12nM PEP005 treatment. Moreover, rapid phosphorylation of Ser643/Ser676 in PKCδ/θ was induced by PEP005 with greater than a two-fold increase as early as 30 minutes post treatment (**Fig 3A, 3B and 3C**). These findings were further validated in PEP005 treated PBMCs from healthy donors. To investigate the involvement of the up-stream kinases in PKC-NF-κB signaling, Western blot analysis was performed using anti-phospho-IκB antibodies. Our data showed that PEP005 treatment induced phosphorylation of IκBα and IκBε, but not of IκBβ (**Fig 3A and 3B**). Interestingly, expression of NF-κB/p65 did not change in the presence of PEP005 (**Fig 3A)**. This is clearly different from the effects of IngB treatment which involved an increased expression of NF-κB/p65 protein [24]. To further confirm the role of PKC-NF-κB signaling in reactivation of HIV latency, J-Lat A1 cells were treated with a PKCθ/δ inhibitor (**Fig 4A)**. Our data showed that inhibition of PKCθ/δ resulted in a reduction of latent HIV reactivation by more than 65%. The addition of the NF-κB inhibitor, Bay-11-7082, to J-Lat A1 cells resulted in an approximately 50% reduction in PEP005-induced disruption of HIV latency (**Fig 4B**). To determine whether PEP005 reactivates latent HIV by promoting NFκB/p65 binding to the HIV LTR, ChIP-qPCR assays were performed with J-Lat A1 cells treated with 12 nM of PEP005 with or without PKCδ/θ inhibitor. PEP005 treatment resulted in a 6-fold increase in NF-κB/p65 binding to HIV LTR region (**Fig 4C)**. This increase was reduced by more than 70% following the addition of PKC inhibitor. Collectively, our findings indicate that PEP005-induced reactivation of latent HIV most likely occurs through the PKCδ/θ-NF-κB signaling pathway. However, this does not exclude a possibility that other PKC isoforms are also potentially involved.

### PEP005 displays minimal toxicity in primary CD4+ T cells

**Fig 5:**
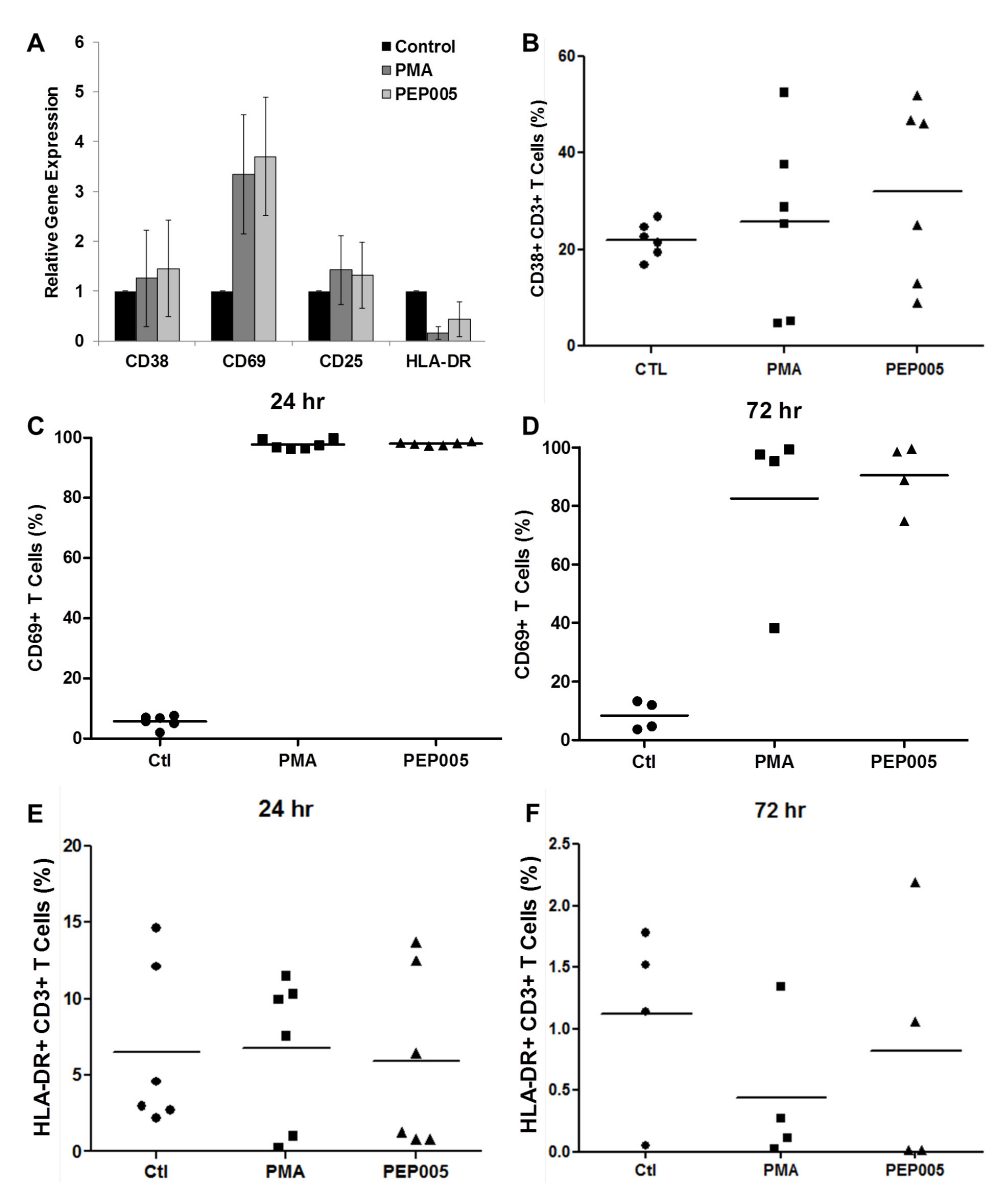
Expression of T cell activation markers in PEP005-treated primary CD4+ T cells. **(A)** CD4+ T cells were isolated from uninfected control subjects and treated with 12 nM PEP005 for 24hrs. Total RNA was extracted, and gene expression of CD38, CD69, CD25, or HLA-DR was analyzed by RT-qPCR. (**B-F**) PBMCs isolated from peripheral blood of healthy HIV-negative donors were treated for 24 or 72 hours with 12 nM of PEP005, and the expression of CD38 **(B)**, CD69 **(C, D)**, and HLA-DR **(E, F)** was evaluated using flow cytometry after co-staining with the CD3 T cell marker.

To be clinically applicable, effective LRAs should be highly potent, minimally cytotoxic and able to penetrate anatomic sanctuaries and immune cell types without inducing global T cell activation [22]. Therefore, we sought to examine the effects of PEP005 on T cell activation and cytotoxicity. Evaluation of the expression of T cell activation biomarkers by RT-qPCR showed that PEP005 treatment did not cause any significant change in the expression of CD38, CD25, or HLA-DR in purified primary CD4+ T cells (**Fig 5A)**. However, there was an increased expression of CD69 in CD4+ T cells. Flow cytometric analysis of global T cells for the expression of CD38, CD69, or HLA-DR further supported the gene expression data. There was no significant change in the expression of CD38 (24 hr) and HLA-DR in CD4+ T cells (**Fig 5B, 5E and 5F**). However, there was an increase in expression of CD69, an inducible glycoprotein that is expressed early during T lymphocytes activation (**Fig 5C and 5D**). These findings provide additional evidence that PEP005 reverses HIV latency through PKC-NF-κB signaling *in vivo*. Since the expression of CD69 is dependent on NF-κB binding to its promoter region, it is understandable that PEP005 induced PKC-NF-κB signaling would up-regulate CD69 expression in CD4+ T cells [44].

**Fig 6:**
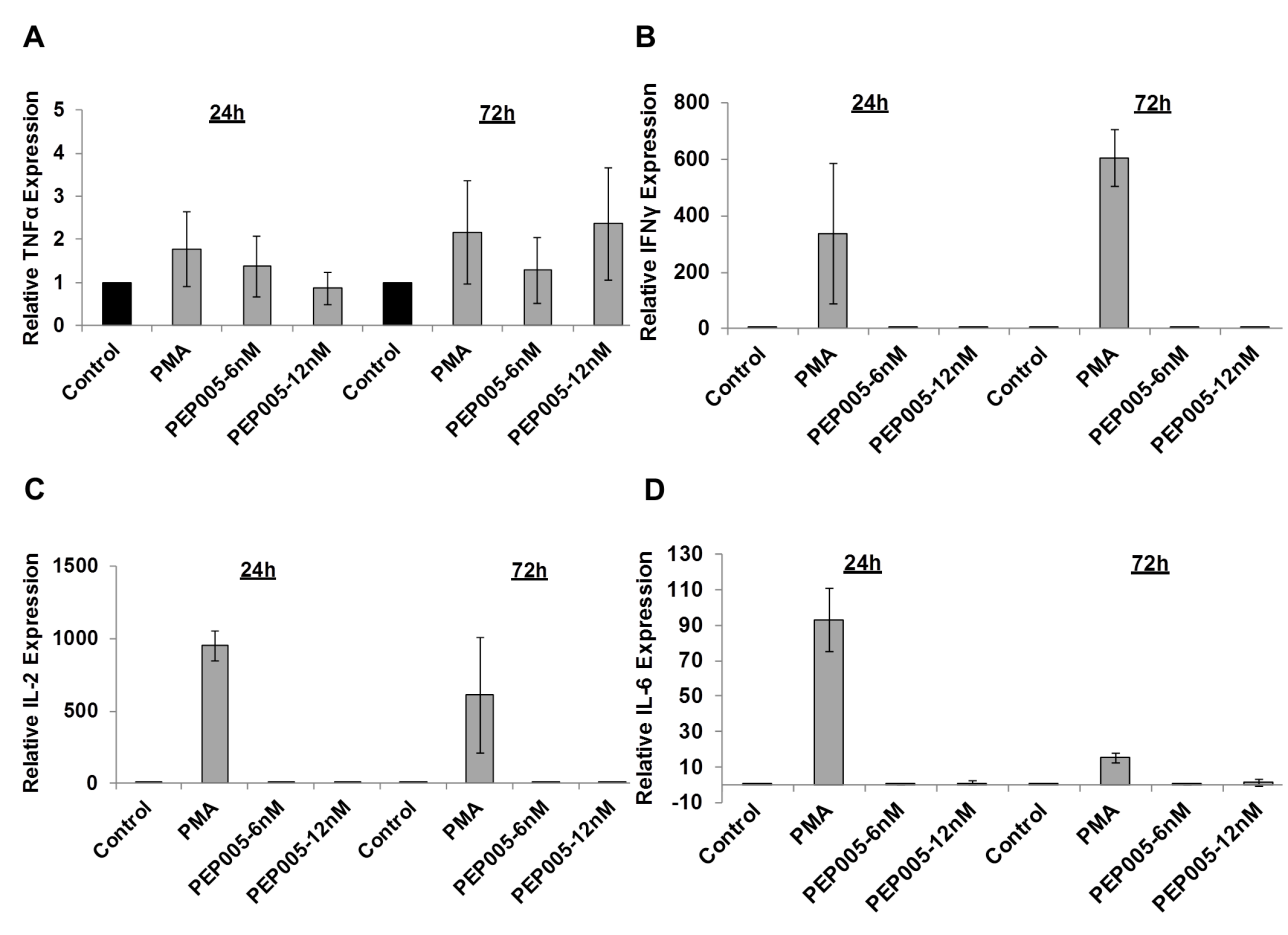
PEP005 does not induce expression of pro-inflammatory cytokines in primary CD4+ T cells from peripheral blood of HIV-negative donors. CD4+ T cells were isolated from healthy donors and treated with 6 or 12 nM of PEP005 for 24 or 72 hours, and the relative expression of TNF-α **(A)**, IFN-γ **(B)**, IL-2 **(C)**, and IL-6 **(D)** was quantified using RT-qPCR and normalized to GAPDH internal control.

We examined the potential side effects of PEP005 on the inflammatory cytokine expression as a possible consequence of increased CD69 production. It is well recognized that acutely or chronically HIV infected individuals seem to express higher levels of inflammatory cytokines including IL-6 and TNF-α in the peripheral blood [45–47]. Therefore, it is important that the agents for disrupting HIV latency do not exacerbate the unresolved chronic immune activation and inflammatory cytokine expression during HIV eradication interventions. To address this question, we examined CD4+ T cells purified from PBMCs of healthy HIV-negative donors *ex vivo* for pro-inflammatory cytokine expression following stimulation with PEP005 for 24 or 72 hours. The expression levels of TNF-α, IFN-γ, IL-2, and IL-6 cytokines were determined by RT-qPCR (**Fig 6A–6D)**. There was no significant increase in the expression of these cytokines, except for TNF-α that showed a tendency towards an up-regulation. However, the increase was not statistically significant.

**Fig 7:**
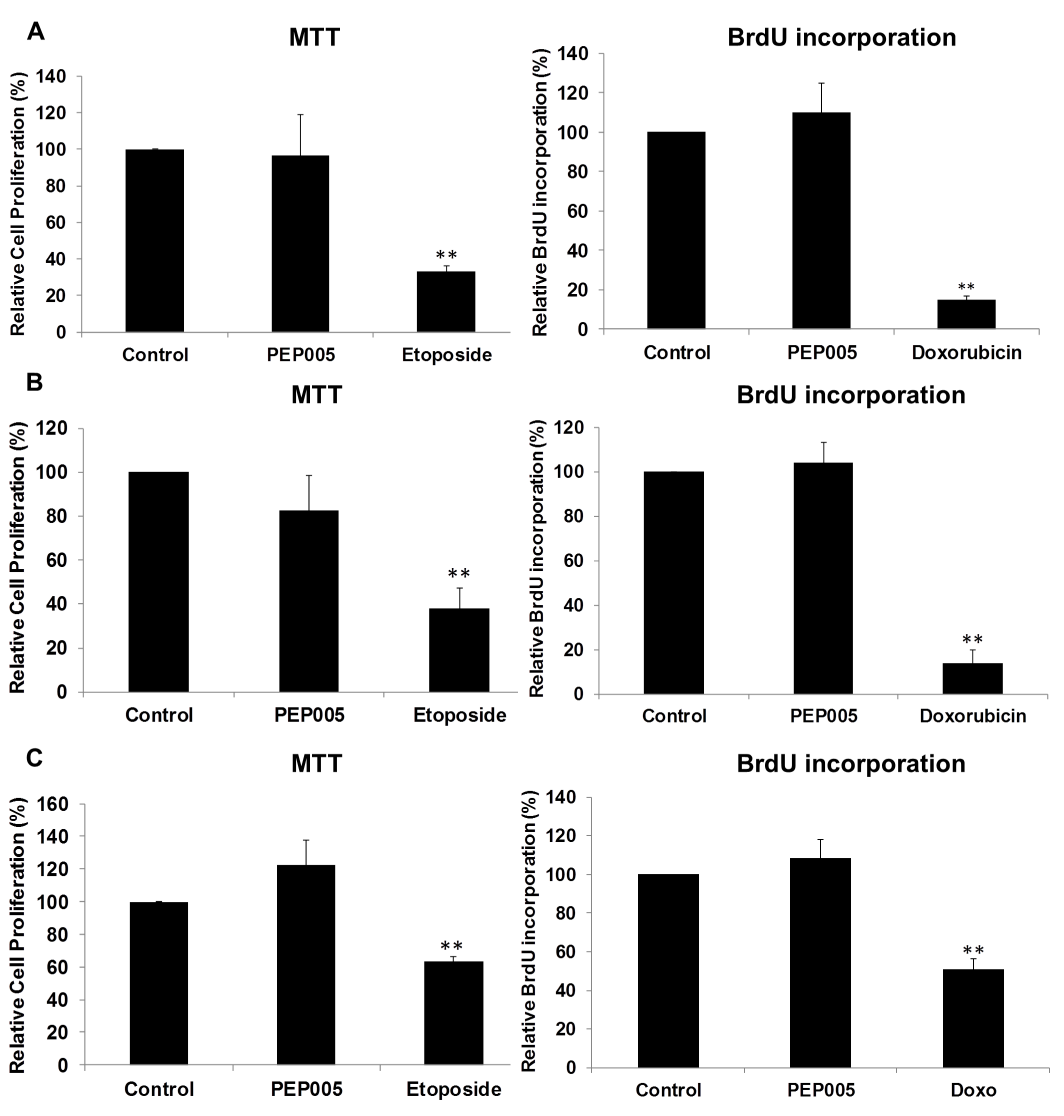
PEP005 causes minimal cytotoxicity and T cell proliferation. To evaluate the impact of PEP005 on the cell viability, U1 cells **(A)**, J-Lat A1 cells **(B)** and CD4+ T cells **(C)** were treated with 12 nM of PEP005 for 24 hours, the cell viability was examined with the MTT assay and the S-phase cell cycle progression was evaluated after BrdU incorporation using BrdU ELISA. 25–50 μM Etoposide or 100–150 μM Doxorubicin (Doxo) served as positive controls for MTT and BrdU assays respectively [48–50]. Statistical analysis was performed in comparison with controls. **, p<0.01.

In order to further evaluate the effects of PEP005 on T cell activation, we determined the ability of PEP005 to induce T cell proliferation and cytotoxicity using BrdU incorporation and MTT assays. PEP005 induced minimal levels of cellular toxicity in both J-Lat A1 and U1 cell lines, as well as in primary CD4+ T cells from peripheral blood samples of healthy HIV-negative donors (**Fig 7**). Importantly, PEP005 treatment did not induce any significant increase in the proportion of cells in S-phase with J-Lat A1 cells, U1 cells or primary CD4+ T cells. In summary, despite the increased CD69 expression, minimal to no induction was found for the expression of pro-inflammatory cytokines and no significant impact was seen on cell cycling, suggesting that PEP005 may be a potential LRA candidate for evaluation *in vivo*.

### PEP005 induces latent HIV expression in primary CD4+ T cells *ex vivo* from individuals with suppressive ART

**Fig 8:**
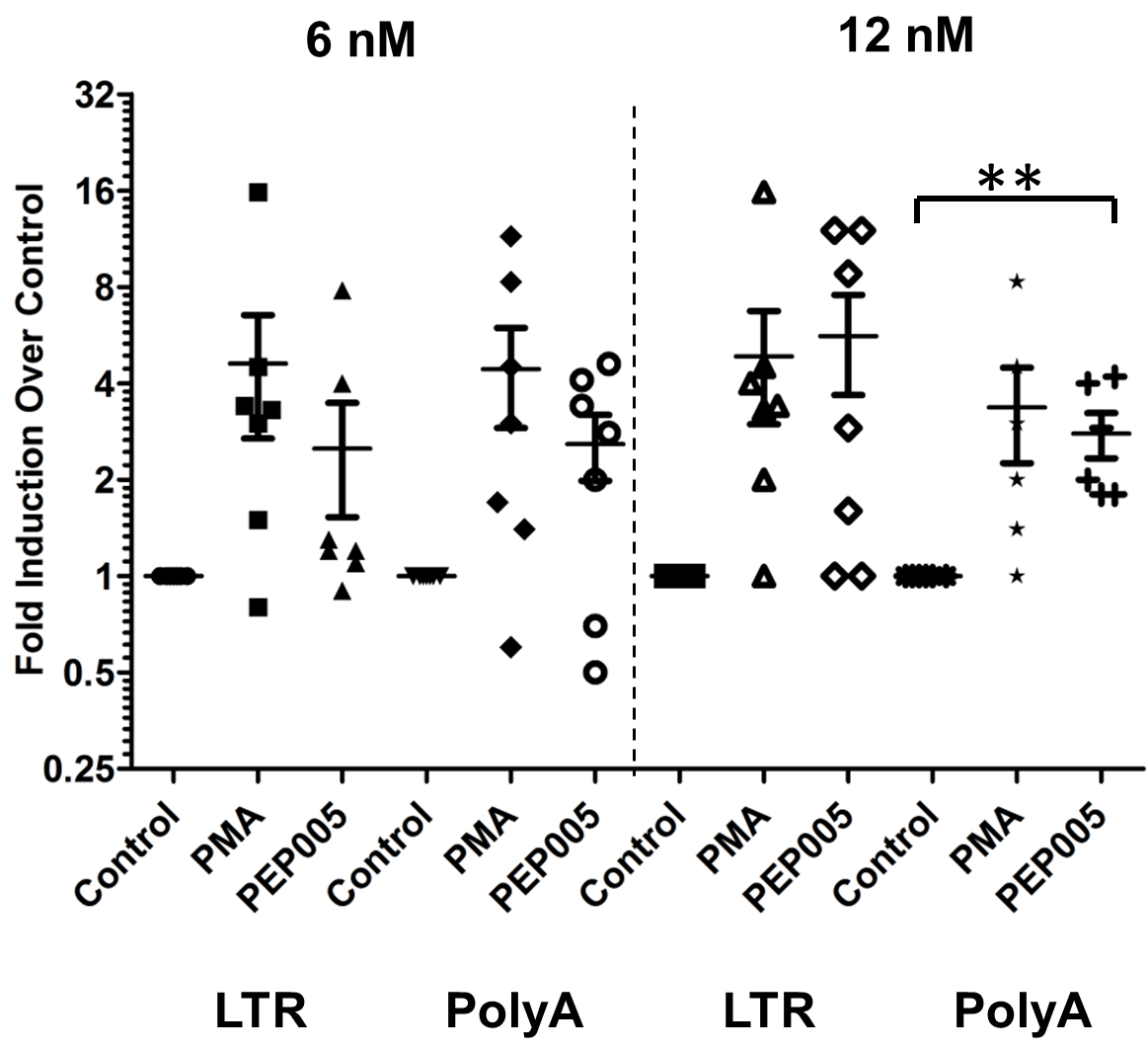
PEP005 induces full-length HIV transcripts in primary CD4+ T cells from HIV infected individuals on suppressive ART. Primary CD4+ T cells were isolated from peripheral blood of HIV infected individuals on suppressive ART and treated with 6 or 12 nM PEP005, 200 ng/ml PMA plus 2 μM Ionomycin, or DMSO for 6 hours. Induction of HIV transcription was measured using RT-qPCR for the 5’ LTR region or Poly A region of the virus. **, p<0.01.

We examined the ability of PEP005 to induce latent HIV expression in primary CD4+ T cells from HIV infected individuals under suppressive ART. The viral loads were below 20 copies/ml of plasma and the CD4+ T cell counts ranged from 260 to 1100 (**Table 1**). Two different doses (6 nM and 12 nM) of PEP005 were added for 6 hrs to purified primary CD4+ T cells from the peripheral blood of these individuals. The level of HIV mRNA expression was measured by RT-qPCR with primers/probe specific for the HIV 5’ LTR region. The magnitude of induction of HIV RNA transcription by PMA we observed in this study differs somewhat from earlier studies [51] because of differences in experimental design (shorter incubation times, PMA concentration) as well as the inherent variability of responses of latently infected primary CD4+ T cells to LRAs. At 6 nM, PEP005 induced an increase in HIV transcription in 2 out of 7 individuals; at 12 nM, HIV transcription was observed in 5 out of 7 individuals (**Fig 8**). To verify the capacity of PEP005 to reactivate latent HIV to produce full-length transcripts, RT-qPCR was performed using the primers/probe targeting HIV 3’ polyadenylation (poly A) region [26]. After 6 hours of PEP005 treatment, 5 of the 7 donors had 2 fold or >2 fold increase in full-length HIV transcripts, while 5 of the 6 donors showed 2 or >2 fold increase after 12 nM of treatment. These results suggest that PEP005 is effective in reactivating transcription of HIV from latently infected cells ex vivo (**Fig 8**). A higher dose of PEP005 is expected to induce more potent reactivation of latent HIV from patient samples.

### HIV induction in the primary CD4+ T cells from HIV infected individuals on ART is enhanced by the combination of PEP005 with JQ1

**Fig 9:**
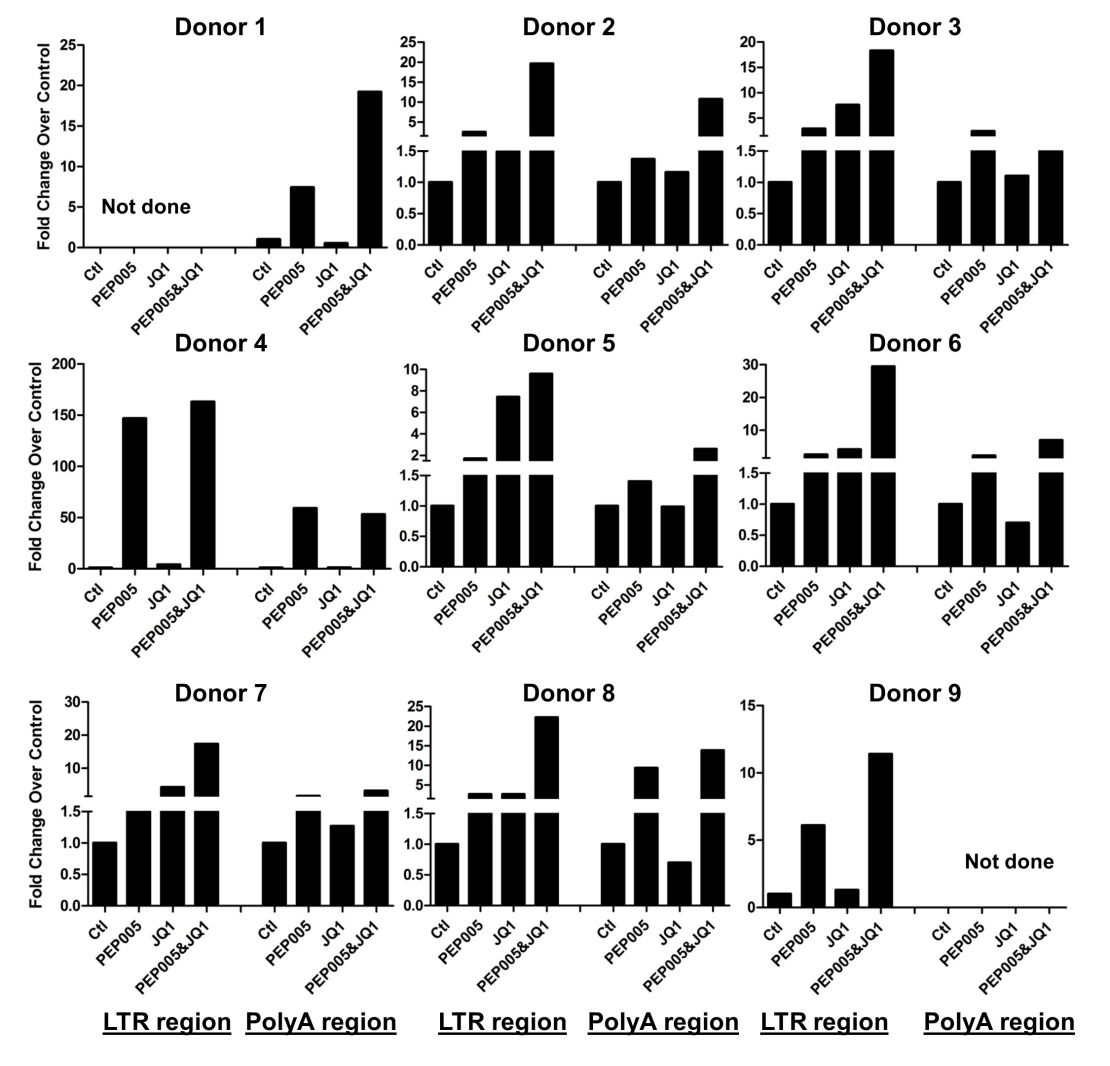
PEP005 and JQ1 synergistically induce HIV transcription 6 hrs after treatment in primary CD4+ T cells from HIV infected individuals on suppressive ART. Primary CD4+ T cells were isolated from the peripheral blood of HIV positive individuals on suppressive ART and treated with 12 nM PEP005 alone or in combination with JQ1 for 6 hours, and HIV transcription was quantified using RT-qPCR for the 5’ LTR region or Poly A region of the virus. The amount of cDNA was only enough to amplify either Poly A region or LTR region during PCR in donor 1 and donor 9.

**Fig 10:**
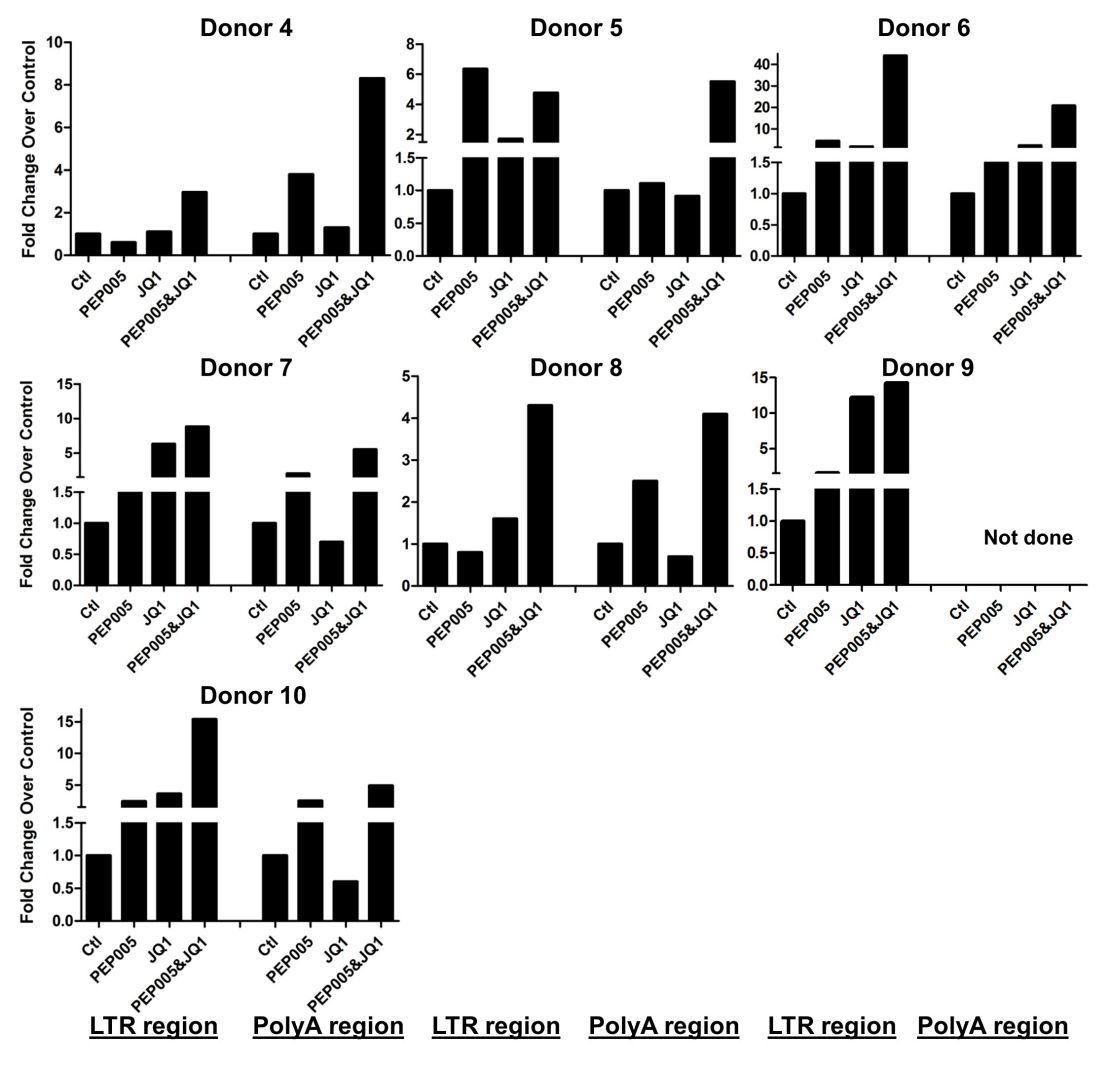
PEP005 and JQ1 synergistically induce HIV transcription 48 hrs after treatment in primary CD4+ T cells from HIV infected individuals on suppressive ART. Primary CD4+ T cells were isolated from the peripheral blood of HIV positive individuals on suppressive ART and treated with 12 nM PEP005 alone or in combination with JQ1 for 48 hours, and HIV transcription was quantified using RT-qPCR for the 5’ LTR region or Poly A region of the virus. The amount of cDNA was only enough to amplify LTR region during PCR in donor 9.

**Fig 11:**
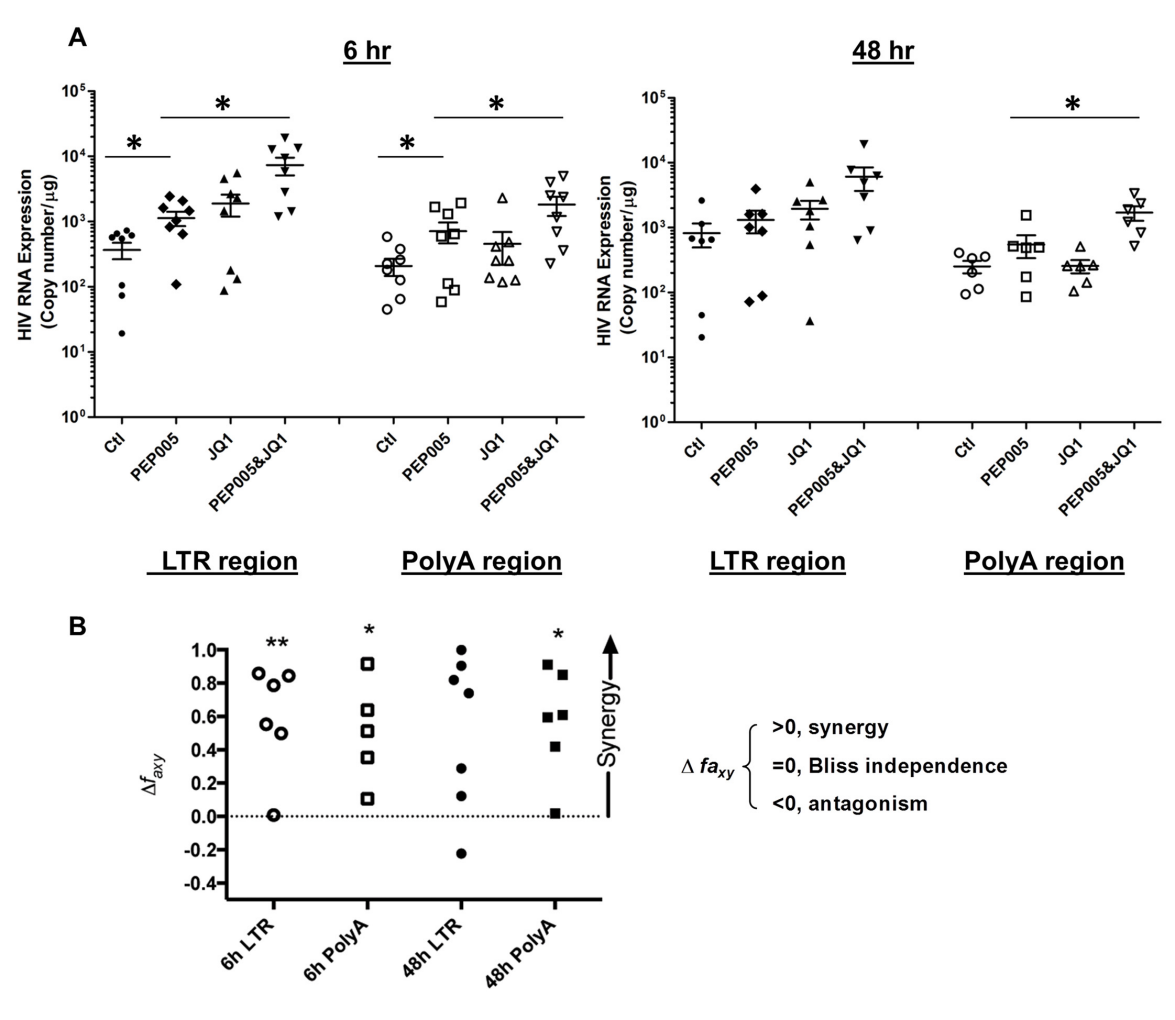
PEP005 and JQ1 synergistically induce HIV transcription in primary CD4+ T cells from HIV infected individuals on suppressive ART. Primary CD4+ T cells were isolated from the peripheral blood of HIV positive individuals on suppressive ART and treated with 12 nM PEP005 alone or in combination with JQ1 for 6 or 48 hours, and HIV transcription was quantified using RT-qPCR for the 5’ LTR region or Poly A region of the virus. **(A)** Copy number of HIV RNA in one μg total RNA in the primary CD4+ T cells after reactivation with 12 nM PEP005. *, p<0.05. **(B)** PEP005 synergizes with JQ1 to significantly increase HIV mRNA expression in primary CD4+ T cells isolated from patients under suppressive ART. The Bliss independence model was utilized for calculation of synergy for LRA combinations [35] (See materials and method). Dotted horizontal line signifies pure additive effect (∆f_axy_ = 0). For these analyses, we included all data for which every parameter for the synergy analysis was available and excluded individual cases where the parameters were not met. Synergy is defined as ∆f_axy_>0 while ∆f_axy_<0 indicates antagonism. Statistical significance was determined using a one tailed paired ratio t-test comparing predicted and observed drug combination effects. *p < 0.05; **p < 0.01.

Our data showed that PEP005 used in combination with JQ1 resulted in synergic reactivation of HIV expression in both the J-Lat A1 cells and the U1 cell models of HIV latency *in vitro* (**Fig 2**). This prompted us to examine whether the combination of JQ1 and PEP005 would significantly enhance the *ex vivo* induction of latent HIV expression in primary CD4+ T cells from HIV infected individuals on suppressive ART. HIV transcription was measured by RT-qPCR following the treatment of cells with 12 nM PEP005 alone, 2 μM JQ1 alone, or 12 nM PEP005 plus 2 μM JQ1 for 6 hrs or 48 hrs. After 6 hrs of stimulation, a strong combined effect of PEP005 and JQ1 induced transcription of HIV RNA using 5’ LTR assay was seen in CD4+ T cell samples from all individuals except one. When transcripts were measured with assay for the HIV poly A region, an enhanced combination effect was seen in 6 of 8 HIV infected individuals. In donor 1 and donor 9, the amount of cDNA was only sufficient to measure HIV RNA with either the Poly A region or LTR region assay (**Fig 9**). The combination treatment at 48 hrs was similarly more potent compared to PEP005 treatment alone in 6 of 7 patient samples when assessed by assay of HIV 5’ LTR region (up to 10 fold increase) and in all 6 patient samples by assay of the Poly A region of the HIV genome (1.6 to 14 fold increase). In donor 9, the amount of cDNA was only sufficient for the Poly A region assay (**Fig 10**). When analyzed as HIV RNA copy number in 1 μg of total RNA from CD4+ T cells, PEP005 significantly induced latent HIV reactivation compared with control treatment after 6 hr incubation, and combined treatment of PEP005 with JQ1 further significantly induced full-length latent HIV reactivation compared with PEP005 treatment alone after 6 or 48 hr incubation (*, p<0.05; ** p<0.01, **Fig 11A**). Applying the Bliss independence model for combined drug effects, criteria for synergic *ex vivo* reactivation of full length latent HIV transcription by PEP005 and JQ1 were met with the caveat that this assessment does not determine whether these viral transcripts were translated into viral proteins of viral particles (**Fig 11B**). Taken together, PEP005 exerted a synergic effect with JQ1 on reactivation of HIV transcription from latency in both cell line models and from CD4+ T cells obtained from patients on suppressive ART that included expression of fully elongated and processed HIV RNAs.

### PEP005 down-regulates cell surface expression of HIV receptors on primary CD4+ T cells

**Fig 12:**
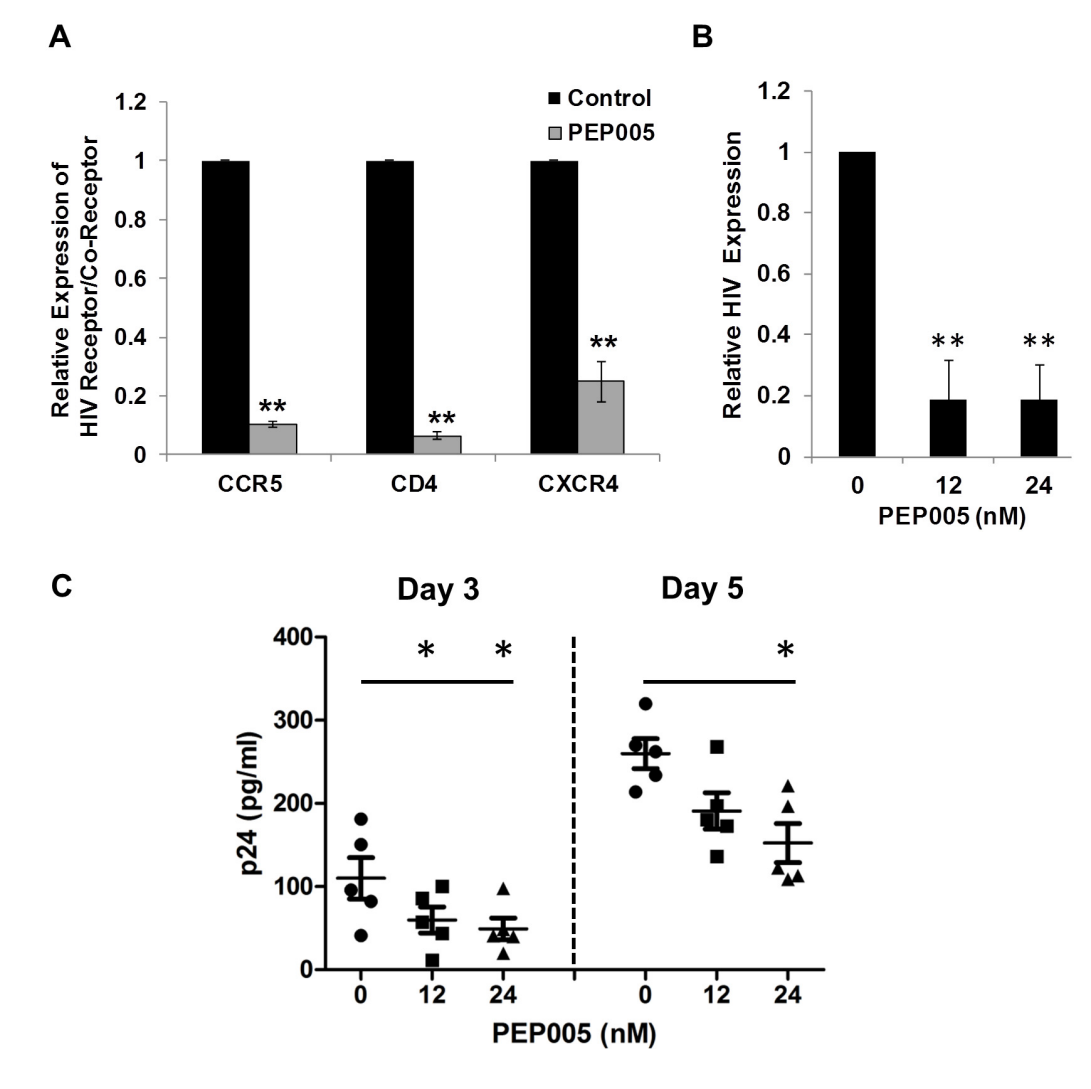
PEP005 down-modulates the expression of CD4, CCR5 and CXCR4 and inhibits HIV infection of primary CD4+ T cells *ex vivo*. **(A)** PEP005 inhibits expression of HIV receptors/co-receptors. The CD4+ T cells were isolated from the peripheral blood of HIV-negative uninfected controls and treated with 12 nM PEP005 for 72 hours. The expression of CD4, CCR5 and CXCR4 genes was evaluated using RT-qPCR after normalizing for GAPDH. **(B)** and **(C)** PEP005 inhibits HIV infection of primary CD4+ T cells *ex vivo*. Primary CD4+ T cells were pre-treated with 12 or 24 nM PEP005 overnight and infected with the virus. The CD4+ T cells were incubated for 5 days without PEP005. The cells were collected for RT-qPCR targeting the HIV LTR region (**B**), or supernatants were collected for p24 ELISA (**C**). *, p<0.05; **, p<0.01.

Recent studies have shown that Prostratin and analogs down-modulate HIV receptor/co-receptor expression, which could have protective effects against the viral infection [52,53]. Conversely, SAHA, known to induce latent HIV expression in HIV infected individuals receiving ART, was reported to increase susceptibility of naive CD4+ T cells to HIV acquisition [54]. Diterpene compounds are known to inhibit expression of HIV receptors/co-receptors including CD4, CCR5, and CXCR4, which are important for the viral attachment and entry into immune cells [27,52,53,55,56]. We sought to examine the effect of PEP005 on the cell surface expression of HIV receptors and co-receptors in CD4+ T cells. Primary CD4+ T cells from peripheral blood samples of healthy HIV-negative donors were treated with 12 nM PEP005 and evaluated for the expression levels of CD4, CCR5, and CCXR4 using RT-qPCR. Our data showed that PEP005 treatment caused a significant reduction in the expression of all these HIV receptors/co-receptors, suggesting that PEP005 may not pose the risk of increasing susceptibility of CD4+ T cells to HIV infection during its reactivation of HIV latency (**Fig 12A)**. Instead, PEP005 contribute to suppression of propagating HIV infection of CD4+ T cells following the reactivation of latent HIV. To investigate the potentially protective effects of PEP005, primary CD4+ T cells were infected with HIV with or without pre-treatment of 12 or 24 nM PEP005 for 24 hrs. The virological outcome in the CD4+ T cell cultures *in vitro* was monitored for 5 days. We observed that PEP005 dampened HIV gene expression as well as decreased the level of viral replication as determined at 3 and 5 days after HIV infection of primary CD4+ T cells (**Fig 12B and 12C**). These findings suggest that PEP005 may help prevent HIV infection of naive CD4+ T cells through down-modulation of HIV co-receptor expression (CD4, CXCR4 and CCR5) and that would be beneficial during the process of latent HIV reactivation.

## Discussion

Since the discovery of stable, latent viral reservoirs in HIV-infected individuals in spite of long-term suppressive ART, several studies have been performed to better understand the mechanisms of the establishment of HIV latency as well as to identify crucial steps for the maintenance of the viral latency [5,6,21]. The identification of memory CD4+ T cells, mostly central memory CD4+ T cells, as the major viral reservoir has provided clues for designing novel strategies for HIV eradication [5,57]. Accordingly, a “shock and kill” strategy was proposed to eradicate latent HIV reservoirs [6]. Diterpene compounds are among the potential candidates for disrupting HIV latency. They are more potent than several other LRAs in inducing latent HIV expression in multiple HIV latency models including *in vitro* J-Lat cell lines, primary CD4+ T cell based cell cultures, and resting CD4+ T cells from the peripheral blood of HIV infected individuals [20,24]. Diterpenes can down-modulate the expression of cell surface receptors that are known to mediate HIV attachment and entry and can potentially prevent the spread of viral infection to bystander CD4+ T cells [27,52–54]. Therefore, it will be useful to identify diterpene compounds suitable for HIV cure studies that could reactivate HIV from the latent reservoirs with low to minimal cytotoxicity while preventing new viral infections of CD4+ T cells.

The data from this study show, for the first time, that PEP005, a diterpene compound, can effectively reactivate latent HIV expression *in vitro* and *ex vivo*. We and others have previously reported that an ingenol ester, IngB, is also able to reactivate HIV from latency [24,27]. Both of these compounds showed efficacy in reactivation of HIV latency at low nanomolar levels without exerting apparent cytotoxicity. IngB could induce increased levels of protein expression of NF-κB, P-TEFb, and IFNγ [24,27] while PEP005 does not increase NF-κB protein expression levels. Activation of NF-κB and P-TEFb is essential for HIV reactivation from latency. However, excessive induction of these proteins may potentially be problematic because NF-κB is a master regulator for multiple signaling pathways, and P-TEFb is a general activator of transcriptional elongation, that also contributes to multiple signaling pathways [58,59]. Further studies are warranted to investigate whether IngB or PEP005 alone can induce protein expression of NF-κB or P-TEFb in resting CD4+ T cells, and not just in proliferating CD4+ T cells. Thirdly, IngB induced IFNγ protein expression [24,27] while PEP005 barely caused any increase in this or other inflammatory cytokine expression.

Although PEP005 shares a similar core structure with IngB, its C3 side chain structure is different and quite distinct from IngB (**Fig 1**). Substitution of the C3-ester by different aliphatic and aromatic side chains confer markedly different biological properties [60,61], so it is likely that differences in molecular mechanisms of action and different T cell activation properties between PEP005 and Ingenol B are conferred by the structural differences of the C3-R. Consequently, PEP005 may offer several advantages over IngB. High levels of ingenols, up to 20 μM, induced NF-κB-independent cell death in Jurkat cells [62]. This concentration is about 1000-fold higher than the PEP005 concentrations used in this study, suggesting that low concentrations of PEP005 capable of HIV reactivation may trigger signaling pathways differently and result in its low-to-minimal cytotoxicity. Therefore, the use of PEP005 at low concentrations for reactivation of latent HIV will be important to minimize cytotoxic effects.

In the recently FDA-approved drug PICATO, PEP005 is the only active component with PKC agonist activity. Pharmacokinetic studies of PEP005 have been performed in small animals *in vivo*. Similar to the safety of the topical application of PICATO, the systemic (intravenous) use of PEP005 in small animals (mini pig and rat model) was reported to be relatively safe, with the maximum nonlethal dose >73 μg/kg (See Assessment report of PICATO to European Medicines Agency, Sept 20, 2012). While additional safety data with systemic administration in non-human primates is needed, these existing data support further investigation of PEP005 as a potential candidate in HIV cure studies.

Since multiple molecular signaling pathways are involved in establishment and maintenance of HIV latency, a single LRA may not be adequate to achieve disruption of the multi-pronged regulatory mechanisms promoting HIV latency. SAHA, an HDAC inhibitor, shows some ability to reactivate HIV. However, the efficacy of SAHA varies among HIV infected individuals indicating that SAHA alone may not be sufficient for effective reactivation of latent HIV for all HIV infected individuals [16,17,63]. Therefore, a combination of compounds should be explored to induce viral expression from latently infected cells [43]. Our data show that PEP005 alone potently reactivates latent HIV expression *in vitro*. However, a combination of PEP005 and JQ1 was substantially more effective in inducing latent HIV in J-Lat A1 cells or U1 cells and in *ex vivo* primary CD4+ T cells from infected patients receiving suppressive ART. This could be attributed to potentiating effects of the PKC-NF-kB pathway on p-TEFb mechanism for reactivating. Future investigations are warranted to determine the range of drug concentrations that will provide the best drug synergy by using PEP005 [64].

Synergistic reactivation of latent HIV also are achieved by combining JQ1 with other ingenol compounds as we previously observed that JQ1 boosted the reversal of HIV latency by IngB in J-Lat A1 cells [24]. However, it is not known whether this combined treatment leads to a synergistic effect on HIV expression in patient cells under ART *ex vivo*. Recently, Sillicano and colleagues showed that PKC agonists, such as Prostratin or Bryostatin 1 had a synergic effect with JQ1 on the reactivation latent HIV *ex vivo* [35]. It was intriguing to note that GSK343, an EZH2 inhibitor, had no capacity to reactivate latent HIV by itself, but displayed increased potency in combination with PEP005 in J-Lat A1 cells. These observations are complemented by a recently published study showing that GSK343 boosts SAHA or JQ1 reactivation of latent HIV in a primary resting T-cell model of HIV latency [65]. These findings suggest that pre-disruption of methylation of histone tails may facilitate efficient reactivation of latent HIV in a combination with a second LRA.

PEP005 displayed potent activity to reactivate HIV from latency, producing polyadenylated viral transcripts while exerting minimal to low cytotoxicity and in the absence of major global T cell activation. Until now, only Bryostatin and PMA have been shown to stimulate comparable transcription of polyadenylated HIV RNA in primary CD4+ T cells isolated from HIV infected individuals on suppressive ART [26]. Considering that PEP005 had a similar capacity as PMA to reactivate latent HIV both *in vitro* and *ex vivo* (**Figs 1D** and **8**), and that PEP005 could induce GFP protein expression in J-Lat A1 cells (**Fig 1**), it seems reasonable to consider that PEP005 as a potential candidate for inducing HIV virus expression *in vivo*. Further *ex vivo* investigations to confirm this using virus outgrowth assays are warranted. There was an indication that PEP005 up-regulated TNFα to some extent but these effects were not significantly different compared to controls. Similar to what is found with other PKC agonists, PEP005-induced NF-kB activation leads to increased expression of CD69 since its promoter contains multiple NF-κB binding sites [44]. However, it was interesting to note that cell proliferation was not significantly altered in J-Lat A1, U1 cells or primary CD4+ T cells, and inflammatory cytokine expression was not significantly enhanced by PEP005. These findings suggest that modulation of some of the T-cell activation markers may not necessarily lead to the global T-cell activation. It has also been proposed that a low level of T cell activation may be required for efficient reactivation of HIV latency [26]. The ability of PEP005 to reduce cell surface expression of CD4, CCR5, and CXCR4 on T cells directly impacts susceptibility of primary CD4+ T cells to *in vitro* HIV infection. This supports the concept that reactivation of latent HIV by PEP005 may block viral spread to uninfected bystander CD4+ T cells, making it an attractive potential candidate for advancing to clinical HIV cure studies. Our study in combination with previous reports suggests that ingenols, including PEP005, represent a new group of lead compounds for combating HIV latency for viral eradication that deserve further study.
